# Low Activation of CD8^+^ T Cells in response to Viral Peptides in Mexican Patients with Severe Dengue

**DOI:** 10.1155/2022/9967594

**Published:** 2022-03-25

**Authors:** Tania Estrada-Jiménez, Lilian Flores-Mendoza, Laura Ávila-Jiménez, Carlos Francisco Vázquez-Rodríguez, Gilma Guadalupe Sánchez-Burgos, Verónica Vallejo-Ruiz, Julio Reyes-Leyva

**Affiliations:** ^1^Centro de Investigación Biomédica de Oriente, HGZ5, Instituto Mexicano del Seguro Social, Km 4.5 Carretera Atlixco-Metepec, CP 74360 Metepec, Puebla, Mexico; ^2^Facultad de Medicina, Decanato de Ciencias Médicas, Universidad Popular Autónoma del Estado de Puebla, 21 Sur 1103, Puebla, CP 72410 Puebla, Mexico; ^3^Departamento de Ciencias Químico Biológicas y Agropecuarias, División de Ciencias e Ingeniería, Universidad de Sonora, Unidad Regional Sur, C.P. 85880 Navojoa, Sonora, Mexico; ^4^Coordinación Auxiliar de Investigación en Salud, Morelos, Instituto Mexicano del Seguro Social, Boulevard Juárez No. 18, Col. Centro C.P., 62000 Cuernavaca, Morelos, Mexico; ^5^Coordinación Auxiliar de Investigación en Salud, Veracruz Sur, Instituto Mexicano del Seguro Social, Calle Poniente 7 # 1350, Col. Centro, C.P., 94300 Orizaba, Veracruz, Mexico; ^6^Unidad de Investigación Médica Yucatán, Instituto Mexicano del Seguro Social, Mérida, Yucatán, Mexico; ^7^Posgrado en Ciencias Químicas, Facultad de Ciencias Químicas, Benemérita Universidad Autónoma de Puebla, 18 Sur y Avenida San Claudio, Colonia San Manuel, 72570 Puebla, Puebla, Mexico

## Abstract

It is acknowledged that antiviral immune response contributes to dengue immunopathogenesis. To identify immunological markers that distinguish dengue fever (DF) and dengue hemorrhagic fever (DHF), 113 patients with confirmed dengue infection were analyzed at 6 or 7 days after fever onset. Peripheral blood mononuclear cells (PBMC) were isolated, lymphocyte subsets and activation biomarkers were identified by flow cytometry, and differentiation of T helper (Th) lymphocytes was achieved by the relative expression analysis of *T-bet* (Th1), *GATA-3* (Th2), *ROR-γ* (Th17), and *FOXP-3* (T regulatory) transcription factors quantified by real-time PCR. CD8^+^, CD40L^+^, and CD45^+^ cells show higher numbers in DF compared to DHF patients, whereas CD4^+^, CD19^+^, and CD25^+^ cells show higher numbers in DHF than DF patients. High expression of GATA-3 accompanied by low expression of T-bet indicates predominance of Th2 response. In addition, higher expression of *FOXP-3* and reduced functional cytotoxic T cells (CD8^+^perforin^+^) were observed in DHF patients. In further experiments, PBMC were stimulated ex vivo with dengue virus E, NS3, NS4, and NS5 peptides, and proliferating T cell subsets were determined. Lower proliferative responses to NS3 and NS4 peptides and reduced CD8^+^ cytotoxic T cells were observed in DHF patients. Our results suggest that immune response to dengue is dysregulated with predominance of CD4^+^ T cells, low activation of Th1 cells, and downregulation of the antiviral cytotoxic activity during severe dengue, likely induced by regulatory T cells.

## 1. Introduction

Dengue is one of the most important viral diseases worldwide and frequently causes outbreaks of high morbidity and economic impact in underdeveloped countries [1, 2]. Dengue virus (DENV) belongs to the Flaviviridae family, genus *Flavivirus*; it is transmitted by mosquitos of the *Aedes aegypti* and *Aedes albopictus* species. DENV possesses an RNA genome that encodes a polyprotein that is posttranslationally processed into three structural proteins (capsid (C), precursor membrane (prM), and envelope (E)) and seven nonstructural (NS) proteins (NS1, NS2A, NS2B, NS3, NS4A, NS4B, and NS5) [3]. There are four DENV serotypes (DENV-1, DENV-2, DENV-3, and DENV-4) [2].

The global incidence of dengue has shown important changes in recent decades; the World Health Organization (WHO) estimates there are 100-400 million dengue infections each year [4]. Reported number of dengue cases increased more than 8-fold in the last two decades, from 505,430 cases in 2000 to 5.2 million in 2019. Reported deaths also increased from 960 to 4032 between the year 2000 and 2015 [4]. For the Pan American Health Organization (PAHO), the largest epidemics recorded in the Region of the Americas occurred in 2015 and 2019, with more than 2.7 million dengue cases reported in 2019 [5]. In particular, Mexico had 213,822 dengue cases in 2019 [5]; Torres-Galicia et al. reported a notable increase in the incidence of DHF in Mexico since the previous decade [6].

Dengue has a wide spectrum of clinical manifestations starting with fever and a mild flu-like syndrome [7]. Febrile phase usually lasts from 2 to 7 days; fever can rise to more than 40°C accompanied by other symptoms such as intense headache, retroocular pain, myalgia, arthralgia, nausea, and rash. Patients can spontaneously recover or progress to severe dengue, which includes DHF and dengue shock syndrome (DSS). DHF is characterized by coagulopathy, increased vascular fragility, and permeability. A patient can enter the critical phase about 3-7 days after fever onset. At this time, fever is dropping (below 38°C) but signs associated with severe disease can manifest, such as intense bleeding, plasma loss, and led in some cases to organ failure and potentially fatal complications [4, 7]. Currently, the WHO classifies dengue into 2 major categories: dengue (with or without warning signs) and severe dengue, but in medical practice, it still used the 1997 classification in DF and DHF [4, 8].

Evolution of febrile to severe dengue seems to be correlated with establishment of the immune response in both primary and secondary infections. Altered immune processes include proliferation of dysfunctional effector T cells induced by cross-reactivity between DENV serotypes, antibody-dependent enhancement (ADE) of infection that increases virus replication in phagocytic cells, dysregulation of complement and coagulation cascades, and overproduction of cytokines, chemokines, and other mediators of inflammation that contribute to clinical manifestations of severe disease [2, 9–11].

Diverse immune cells are targets of DENV infection, including monocytes/macrophages and dendritic cells [2]. Early after infection, these cells contribute to establish a proinflammatory state by overproduction of several cytokines such as TNF-*α*, IL-1*β*, IL-2, IL-6, and type I IFNs. This leads to activation of CD4^+^ Th2 cells that produce IL-2, IL-4, IL-5, and IL-13 that consolidate the inflammatory process, as well as IL-10 and TGF-*β* that induce differentiation of regulatory T cells (Treg) and contribute to downregulate the antiviral response [2, 11–15]. In contraposition, IL-12 and IFN-*γ* induce activation of Th1 cells that promote activation of CD8^+^ cells and the cytotoxic antiviral response necessary to clear out infection [12]. Diverse transcription factors are the main regulators of T cell differentiation; they control the type of cytokines secreted and the route that will follow the immune response; indeed T-bet, GATA-3, ROR*-γ*, and FOXP-3 are involved in the control of the differentiation process of Th1, Th2, Th17, and Treg cells, respectively [16, 17].

The protective role of T cells in dengue is controversial; some reports showed that early activation of CD8^+^ T cells is crucial to restrict DENV infection [18, 19]; indeed, higher proliferation and cytotoxic activity of CD8^+^ T cells with production of IFN-*γ* have been associated with protection against secondary infections, regardless of DENV serotype [18–22]. In contrast, high levels of CD4^+^ cells were found in severe and fatal dengue cases [21, 23, 24], even with increased numbers of CD8^+^ T cells in patients with DHF [18, 25, 26]. At respect, the proinflammatory response induced by CD4^+^ cells to heterotypic secondary infections seems to be associated with proliferation of low-affinity memory CD8^+^ T lymphocytes that are not as functional as high-affinity memory cells to control DENV infection [24, 25, 27, 28].

Specific T cell response can be induced by different epitopes of structural and nonstructural DENV proteins [13, 14, 20, 29–32]. Kurane and Mathew studied the immune response to attenuated viruses in vaccinees as well as in patients with natural infection in Thailand; they showed that NS3 have multiple antigenic sites recognized by T cells [33, 34]. Rivino et al. evaluated the reactivity of T cells by using a library of overlaid peptides that cover all DENV-2 proteome in adult patients from Singapore that suffer a secondary infection. They found a higher proportion of CD8^+^ T cells induced by NS3 and NS5 peptides; meanwhile, E and C peptides induced CD4^+^ T cells [35]. Tian et al. showed that CD4^+^ T cells were predominantly directed against the capsid protein followed by E, NS3, and NS2 proteins, while the activation of CD8^+^ T cells was induced by NS3, followed by capsid, NS5, and NS4/B proteins [31]. Weiskopf et al. made a complete analysis of CD8^+^ T cells in a hyperendemic region of Sri Lanka. They measured the response of IFN-*γ-*producing cells ex vivo; the most antigenic proteins were NS3, NS4B, and NS5 [20]. Other authors showed that induction of CD4^+^ or CD8^+^ T cells depends on dengue virus serotype and the patient's HLA haplotypes [32, 33]. Thus, genetic factors and geographic location of infected people have an important influence on the specificity and intensity of cellular immune response and its role in protection [15, 20, 25, 28, 36, 37].

In this work, cellular parameters of the immune response were examined in patients during a dengue outbreak that occurred at the central region of Mexico, phenotyping of T cells and their transcription factors, and the proliferative response of immune cells induced by dengue peptides were correlated with disease severity.

## 2. Materials and Methods

### 2.1. Ethical Statement

This study was conducted in accordance with international ethical principles and the Declaration of Helsinki (last update Brazil 2013). The research protocol was approved by the Committee of Ethics in Health Research of the Mexican Institute of Social Security (IMSS) and recorded under registration numbers R-2011-2103-29 and R-2012-2104-2. All patients (and their relatives) were informed about the study, and written consent to participate was obtained from each participant. Parents or legal tutors authorized the participation and signed informed consent of patients lower than 18 years old. Patients' confidentiality was assured by assigning a progressive study number to their data and blood samples.

### 2.2. Clinical Procedures

The study was done with patients admitted at IMSS “HGZ5” General Hospital located at Metepec, Puebla, Mexico. Healthy blood donors from a geographical zone free of dengue were enrolled at IMSS High Specialty Medical Unit located at Puebla City. Confirmatory dengue diagnosis was done by means of IgM and IgG ELISA kits (PanBio Diagnostic) and/or detection of NS1 antigen (Platelia Bio-Rad). The 1997 WHO classification of dengue, still in practice at this hospital, was used to classify cases into DF and DHF [9].

Blood samples were obtained from all patients and submitted to hematological and biochemical laboratory tests, including hematic biometry, platelet and differential leukocyte counts, and the concentrations of hepatic enzymes aspartate (AST) and alanine (ALT) aminotransferases. Other tests such as abdominal ultrasounds were requested for medical purposes only.

For immunological tests, 3 to 5 ml blood samples were obtained in heparinized tubes at 6 or 7 days after fever onset. Samples were centrifuged at 1700 rpm at 4°C for 7 minutes; plasma was separated and stored in aliquots at -70°C until use. Ex vivo PBMC were separated by Ficoll-Histopaque density gradient centrifugation and analyzed by flow cytometry. Their mRNA was isolated for qPCR assays.

### 2.3. Flow Cytometry

The following monoclonal antibodies were used for flow cytometry: CD3-FITC (clone UCHT1), CD4-APC (clone OKT-4), CD8-APC-Cy7 (clone SK1), CD19-PE (clone HIB19), CD69-PE (clone FN-50), CD45-PE (clone UCHC1), CD40L-PE (clone 24-31), and CD25-PE (clone PC61.5) all from eBioscience (currently Thermo Fisher Scientific, Waltham, MA, USA). Anti-perforin antibody (clone dG9, sc-33655) and anti-IgG2b-PE (both of Santa Cruz Biotechnology, Dallas TX, USA) were used for intracellular staining. All antibodies used in multiparametric flow cytometry were tested and conditions calibrated before their use in samples.

Ex vivo PBMC were thawed and resuspended at 1 × 10^6^ cells/ml, and lymphocyte subsets were identified by flow cytometry using the BD FACSCanto II Flow Cytometry System and the FACSDiva Software (BD Biosciences, San Jose CA, USA). Bicolor panels were analyzed as follows: for helper T cells, CD3/CD4; for cytotoxic T cells, CD3/CD8; for B cells, CD3(-)/CD19; and for activated T cells, CD3 coupled with either CD69, CD45, CD40L, or CD25.

### 2.4. T Cell Proliferation Assays

Peptides of dengue virus NS3, NS4, NS5, and E proteins [38–40] were synthesized by GenScript (NJ, USA) at >90% purity. The peptide sequences and characteristics are shown in Table 1. Peptides were reconstituted at 10 mg/ml following the manufacturer's specifications: P1, P2, P4, P5, P6, P8, P9, P12, P13, and P14 in 10% DMSO in water, while P3, P7, and P11 peptides in 3% ammonia in water. Peptides were stored at -20°C until use. Mixtures of peptides corresponding to each viral protein were prepared as follows: E (P1, P2, and P3), NS3 (P4, P5, and P6), NS4 (P7, P8, P9, and P10), and NS5 (P11, P12, P13, and P14). The final concentration of each peptide in a mixture was 10 *μ*g/ml.

For microplate priming, 96-well microplates were incubated overnight with 1 *μ*g/ml suspensions of monoclonal antibodies with functional assay activity CD28 (clone 37.51) and CD3 (clone HIT3a) both of eBioscience, which induce costimulatory signals that increase the response to synthetic peptides [41]. The next day, PBMC of patients and controls (1.8 × 10^6^ cells) were labelled with 5 *μ*M CFSE (eBioscience) 5 minutes at 37°C in centrifuge tubes. After that, cells were washed with PBS and resuspended in RPMI 1640 containing 10% FBS, 1% HEPES, 1% L-glutamine, and 1% penicillin-streptomycin. Then, 1 × 10^5^ PBMC were added to each well of a primed microplate and stimulated with the mixture of peptides mentioned previously (E, NS3, NS4, or NS5). All experiments were done by triplicate. DMSO (10%) and ammonia (3%) solutions in conditioned RPMI medium without peptides were used as negative controls. Cells stimulated with Phaseolus vulgaris hemagglutinin (PHA, SIGMA, 20 ng/ml) were included as positive proliferation controls. Microplates were incubated at 37°C, 5% CO_2_ for 120 hours. After that time, proliferation of CD4^+^ and CD8^+^ cells was determined by flow cytometry using the BD FACSCanto II Flow Cytometry System and the FACSDiva Software (BD Biosciences, San Jose CA, USA).

### 2.5. Ex Vivo Relative Gene Expression Analysis

Total RNA was extracted directly from ex vivo PBMC using TRIzol (Invitrogen) following manufacturer's instructions. cDNA was synthetized by using random primers and the RevertAid H Minus Reverse Transcriptase Kit (Thermo Fisher Scientific, USA) at 25°C for 10 min, 42°C for 60 min, and 70°C for 10 min. Expression of *T-bet*, *GATA-3*, *ROR-γ*, and *FOXP-3* genes was determined by qPCR using the SYBR Green/ROX-PCR Master Mix (Thermo Fisher Scientific, USA) and the following primers: *T-bet* forward 5′-CAC GCA CTT CCG CAC ATT CC-3′, *T-bet* reverse 5′-TCC AGC AGC TCG AAG AGG CA-3′, GATA-3 forward 5′-ACA ATC TGC CTC AAT CAC TCT G 3′, GATA-3 reverse 5′-TTG ACT TGG ATT GGG ATT TTG-3′, *ROR-γ* forward 5′-GTC CAA CAA TGT GAC CCA G-3′, *ROR-γ* reverse 5′-CTT TCC ACA TGC TGG CTA CA-3′, *FOXP-3* forward 5′-AAG CAG CGG ACA CTC AAT-3′, and *FOXP-3* reverse 5′-AGG TGG CAG GAT GGT TTC-3′. *HPRT* was included as endogenous control in all experiments using the primers *HPRT* forward 5′-CCT GGC GTC GTG ATT AGT GAT GAT-3′ and *HPRT* reverse 5′-CGA GCA AGA CGT TCA GTC CTG TC-3′. All reactions were run in the StepOne Real-Time PCR system (Applied Biosystems). Before the analysis of gene expression in samples, dynamic ranges were calculated and dissociation curves corresponding to each gene were depicted to determine assay specificity. Relative gene expression between the control and patient groups was calculated using the 2^-*ΔΔ*CT^ method, where ∆CT is the difference in the threshold between any target gene (*T-bet*, *GATA-3*, *ROR-γ*, and *FOXP-3*) and the endogenous gene (*HPRT*). Expression of each mRNA transcription factor was arbitrarily assigned a value of 1 in uninfected controls, and relative expression changes were calculated in patients.

### 2.6. Statistical Analysis

Normality of data distribution was tested by the Kolmogorov–Smirnov test. The data were represented as means with standard deviations (SD). The nonparametric Mann–Whitney test, *T* test, or *χ*^2^ test was used to compare independent groups as applicable. All analyses were done in GraphPad Prism v7.0 software; differences between groups were considered significant at *p* < 0.05.

## 3. Results

### 3.1. Demographic Characteristics and Clinical Parameters

According to hospital's archives, 189 patients were admitted with diagnosis of dengue from July 1 to October 1, 2015; of these, 113 patients were included in the study after completing their clinical tests and agreed to participate voluntarily by signing the informed consent. Sixty-three patients presented DF and fifty have DHF. Plasma values of NS1, IgM, and IgG confirmed dengue infection. Dengue serotype was not determined. The median age was 48 and 41 years for DHF and DF patients, respectively, with a variation between 4 and 71 years.  Table 2 summarizes the main characteristics of the study groups.

### 3.2. Clinical Signs

The most frequent signs and symptoms present in dengue patients were fever, headache, back pain, arthralgia, and rash. The hemorrhagic signs were variable; a high number of patients presented petechiae (46/113), epistaxis (35/113), and gingival hemorrhage (16/113). Hematemesis and melena were less frequent (7/113 and 4/113, respectively). Among the 50 patients with DHF, 26 presented both petechiae and epistaxis, and 10 presented simultaneously petechiae, epistaxis, and gingival hemorrhage. One patient with DHF presented ascites.

### 3.3. Hematic Changes

No significant differences were found between groups regarding hematocrit, hemoglobin, creatinine, and the proportion of leukocytes, lymphocytes, monocytes, or neutrophils. Significant differences were found between DF and DHF patients for platelet counts and serum albumin concentrations (see Table 2). Most patients showed platelet values < 10,000 cells/mm^3^ in both DF and DHF patients (6765 ± 2642 and 2624 ± 1830 cells/mm^3^, respectively; *p* < 0.0001). AST and ALT values were higher than the reference in both DF and DHF patients, but only two DHF patients presented values > 1000 UI/ml for both hepatic enzymes (see Table 2).

### 3.4. Lymphocyte Proportions in Dengue Patients

**Figure 1:**
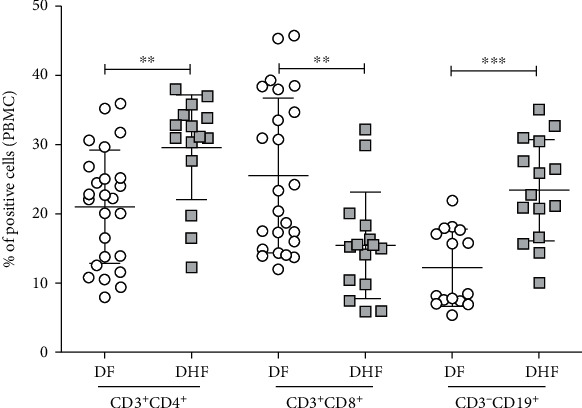
Proportion of CD4^+^ T cells (CD3^+^CD4^+^), cytotoxic T cells (CD3^+^CD8^+^), and B cells (CD3^−^CD19^+^) determined by flow cytometry in ex vivo PBMC of patients with dengue fever (DF) and dengue hemorrhagic fever (DHF). The error bars represent mean ± SD. ^∗∗^*p* < 0.01 and ^∗∗∗^*p* < 0.001 by the Mann–Whitney *U* test.

**Figure 2:**
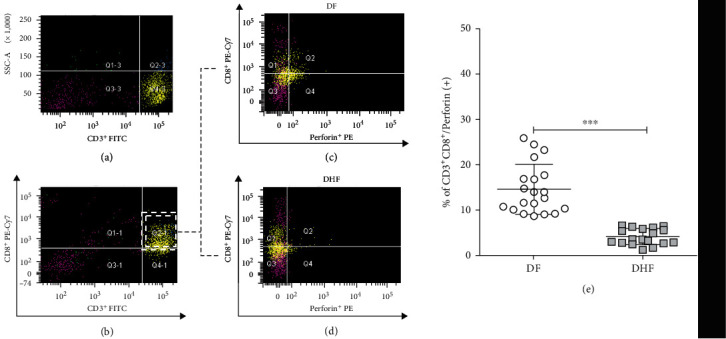
Analysis of functional cytotoxic T lymphocytes in *ex vivo* PBMC of patients with dengue fever (DF) and dengue hemorrhagic fever (DHF) by flow cytometry. Representative plots of the gating strategy for the analysis of CD3^+^/CD8^+^/perforin^+^ T cells (a–d). First, singlets and live cells were selected. (a) CD3^+^ and (b) CD3^+^/CD8^+^ double positive cells were gated and used to determine perforin expression in (c) DF and (d) DHF patients. (e) Percentages of activated CD3^+^CD8^+^perforin^+^ cells in patients with DF (*n* = 23) and DHF patients (*n* = 20). The error bars represent mean ± SD. ^∗∗∗^*p* < 0.001 determined by the Mann–Whitney *U* test.

The proportion of CD3^+^CD4^+^ T cells was higher in DHF than in DF patients(29.59 ± 7.56% and 21.03 ± 8.18%, respectively, *p* = 0.022) (Figure 1). The proportion of B cells (CD3^−^CD19^+^) was almost twofold higher in DHF than in DF patients (23.43 ± 7.32% vs. 12.2 ± 5.59%, *p* < 0.0001). In contrast, a smaller number of CD3^+^CD8^+^ T cells were found in DHF compared to DF patients (15.45 ± 7.67% vs. 25.54 ± 11.19%; *p* = 0.0045) (see Figure 1). In addition, significant reduction of activated cytotoxic T cells (CD3^+^CD8^+^perforin^+^) was found in DHF patients compared to DF patients (4.17 ± 1.75% vs. 14.65 ± 5.47%, respectively; *p* < 0.001) (Figure 2).

**Figure 3:**
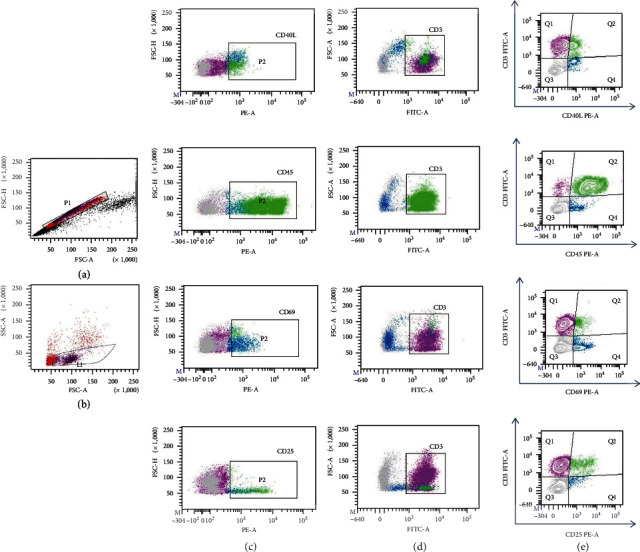
Strategy for the analysis of activation molecules on ex vivo PBMC and CD3^+^ population in patients with dengue fever (DF) and dengue hemorrhagic fever (DHF). (a, b) First, singlets (P1) and live (L1) cells were selected. (c) Activation molecules were identified in the PBMC population (P2): CD40L^+^, CD45^+^, CD69^+^, and CD25^+^ subsets are represented from top to bottom. (d) CD3^+^ cells were gated and used to determine double positive cells. (e) Representative plots of activation molecules (CD40^+^, CD45^+^, CD69^+^, and CD25^+^) expressed in T cells. Q1 represents CD3^+^ cells; Q2 represents double positive cells.

Activation of T cells was analyzed by the identification of CD3 together with CD40L, CD45, CD69, and CD25 markers (Figure 3). Proportions of total CD40L^+^ cells were significantly higher in DF than in DHF patients either in whole PBMC (19.06 ± 13.54 vs. 4.02 ± 2.96) or CD3^+^ cells (3.19 ± 1.98 vs. 1.65 ± 1.29) (*p* = 0.0009 and *p* = 0.0053, respectively) (Table 3). CD45^+^ cells were more abundant in DF than DHF (53.02 ± 16.09 vs. 35.73 ± 10.4; *p* < 0.0004), but they did not differ between T cells. In contrast, CD25 expression was higher in DHF than in DF patients, either analyzing whole PBMC (18.36 ± 8.15 vs. 7.49 ± 3.49%; *p* = 0.0001) or CD3^+^ T cells (3.0 ± 2.02 vs. 1.24 ± 0.81, *p* = 0.0018), respectively. No difference was observed in CD69 expression either in PBMC or T cells.

### 3.5. Relative Expression of T Cell Transcription Factors

**Figure 4:**
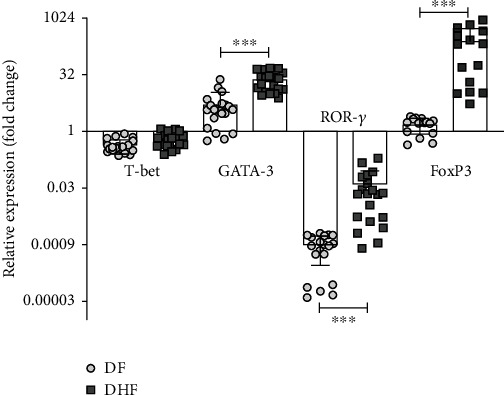
Relative gene expression analysis of T cell transcription factors. The expression of *T-bet* (Th1), *GATA-3* (Th2), *ROR-γ* (Th17), and *FOXP-3* (Treg) was analyzed ex vivo in PBMC of DF and DHF patients; data are expressed as fold increments compared with the expression level of the control group, which was assigned an arbitrary value of 1. Error bars represent mean ± SD. Significant differences ^∗^*p* < 0.05, ^∗∗^*p* < 0.01, and ^∗∗∗^*p* < 0.001 by the Mann–Whitney *U* test.

T-bet (Th1), GATA-3 (Th2), ROR*-γ* (Th17), and FOXP-3 (Treg) transcription factors associated with helper T cell subpopulations were directly analyzed ex vivo in PBMC. Both DF and DHF patients showed low expression of *T-bet* mRNA (0.4 ± 0.17 and 0.6 ± 0.2-fold) which indicates low levels of Th1 cells and negative values of *ROR-γ* expression (0.0009 ± 0.0006 and 0.04 ± 0.05-fold), which suggest downregulation of Th17 cells (Figure 4). The absence of Th17 cells (*ROR-γ*) was corroborated by the lack of IL-17 in the sera of DENV patients (limit of detection 20 pg/ml; data not shown).

In contrast, DENV patients showed higher expression of *GATA-3* and *FOXP-3* mRNA that indicate the presence of Th2 and Treg cells, respectively. *GATA-3* was significantly higher in DHF than in DF patients (24.3 ± 13.8 vs. 4.1 ± 3.5-fold, respectively; *p* < 0.001). *FOXP-3* showed the highest differences in expression between DHF and DF (205 ± 191 vs. 1.8 ± 0.9-fold, respectively; *p* < 0.0001) (Figure 4).

### 3.6. T Cell Response to Viral Peptides

**Figure 5:**
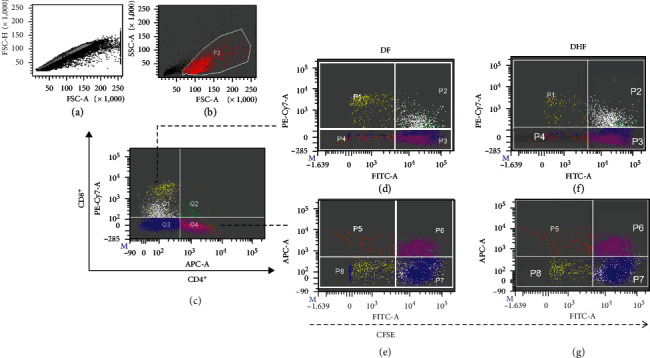
Strategy for the analysis of proliferating T cells labelled with CFSE. Singlets and live cells were selected (a, b). CD4^+^ and CD8^+^ positive cells were defined with APC or PE-Cy7, respectively (c). Representative plots show CD8^+^ and CD4^+^ cells in (d, e) DF and (f, g) DHF patients; P1 and P5 represent CD8^+^ and CD4^+^ proliferating cells (low CFSE), respectively. These data in cells stimulated with dengue virus peptides are shown in Figure 6. P2 and P6 represent CD8^+^ and CD4^+^ nonproliferating cells (high CFSE), respectively.

**Figure 6:**
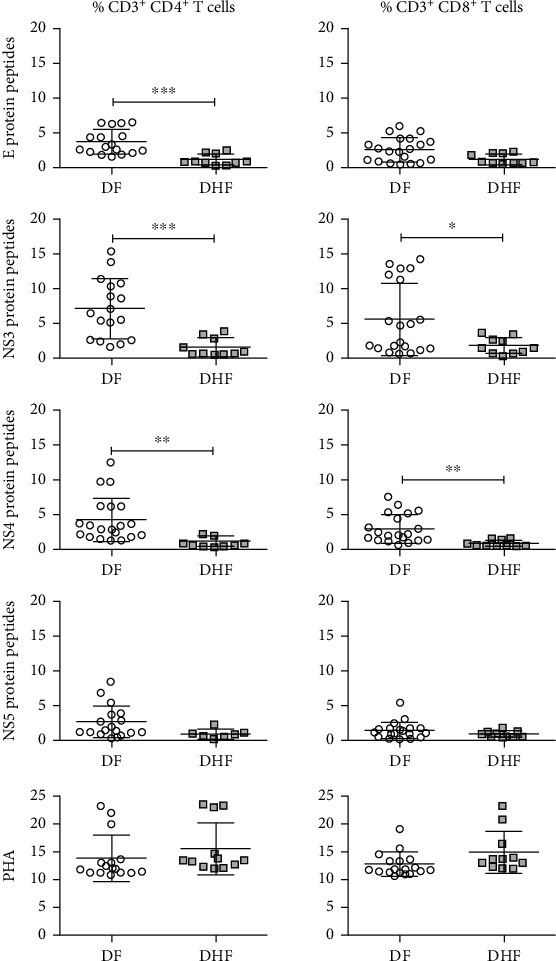
CD4^+^ and CD8^+^ proliferating cells stimulated with dengue virus peptides in dengue fever DF (*n* = 23) or dengue hemorrhagic fever DHF (*n* = 20) patients. Plots show the proportion of CD4^+^ and CD8^+^ cells induced by peptides of E, NS3, NS4, and NS5 viral proteins and the positive control stimulated with PHA. Error bars represent the mean ± SD. Significant differences ^∗^*p* < 0.05, ^∗∗^*p* < 0.01, and ^∗∗∗^*p* < 0.001 by the Mann–Whitney *U* test.

Proliferative responses induced *ex vivo* by viral peptides were analyzed by flow cytometry using CFSE to label T cells. The strategy to analyze CD4 and CD8 proliferating T cells is shown in Figure 5; the objective was to evaluate the relative frequency of proliferating (CFSE low) and nonproliferating (CFSE high) in CD4^+^ and CD8^+^ cells. The proportion of proliferating CD4 and CD8 cells induced by viral peptides in DF and DHF is shown in Figure 6.

In general, T cell responses were higher in DF than in DHF patients; indeed, the proportion of CD4^+^ cells induced by E, NS3, NS4, and NS5 was 3.6 ± 1.8 vs. 1.1 ± 0.8% (*p* = 0.0003), 7.2 ± 4.3 vs. 1.6 ± 1.3% (*p* = 0.0006), 4.3 ± 3.2 vs. 0.9 ± 0.6% (*p* = 0.0032), and 2.6 ± 2.3 vs. 0.8 ± 0.7% (*p* = 0.21), respectively. The proportion of CD8^+^ cells induced by E, NS3, NS4, and NS5 peptides was 2.3 ± 1.7 vs. 1.03 ± 0.7% (*p* = 0.28), 5.7 ± 5.2 vs. 1.8 ± 1.2% (*p* = 0.029), 3.03 ± 2.1 vs. 0.7 ± 0.4% (*p* = 0.0034), and 1.4 ± 1.2 vs. 0.8 ± 0.5% (*p* = 0.21) either in DF or DHF, respectively. NS3 peptides were more immunogenic than the other peptides. Positive control stimulus (PHA) induced proliferation of both CD4^+^ and CD8^+^ T cells (around 15%) in both DF and DHF patients. Negative controls increased less than 0.1% for both groups.

## 4. Discussion

The balance between Th1 and Th2 cells is crucial to orchestrate an immune response that protects against virus infection but maintains homeostasis [42]. Differentiation of CD4^+^ naïve T cells into Th1, Th2, Th17, and Treg cell lineages depends on the expression of T-bet, GATA-3, ROR-*γ*, and FOXP-3 transcription factors, respectively. T-bet and GATA-3 rival each other for activation; GATA-3 prevents the polarization of Th1 and Th17 response by promoting T-bet and ROR-*γ* downregulation and controls the secretion of their related cytokines [16, 43–45]. In this work, we found that patients with dengue did not significantly express *T-bet* and *ROR-γ*, but they showed higher expression of *GATA-3* and *FOXP-3*, particularly in patients that worsen to DHF. Higher expression of GATA-3 is in accordance with its ability to promote the proinflammatory response that is mediated by IL-4, IL-5, IL-6, and IL-13 [16, 46, 47]. Their presence is also indicative of persistent inflammation and severity in other Flavivirus infections and dengue [21, 24, 48, 49].

Increased expression of FOXP-3 in patients with DHF suggests the differentiation of regulatory T cells involved in the secretion of IL-10. According to this, we have previously found high levels of IL-10 in the serum of patients with DHF [50].

Several studies showed that CD8^+^ cells play important roles in the control of DENV infection [18–20]. Our results showed that CD8^+^ cells were more abundant in DF than in DHF patients. In accordance, an increased proportion of IFN-*γ*-producing CD8^+^ T cells has been found in patients with subclinical dengue infections [21, 22]. Other reports showed that CD8^+^ cells contribute to protect against homotypic and heterotypic DENV reinfection [13, 21, 51].

However, it has been proposed that cell immunity might be incompletely activated during the febrile phase of dengue due to an altered cytokine production, decreased CD8^+^ T cell proliferation, and augmented T cell apoptosis [18, 26, 52]. Reduced cytotoxic function has been reported for mild and severe dengue during defervescence [53]. In our study, reduction in functional cytotoxic T cells was revealed by the lower proportion of CD8^+^perforin^+^ cells in DHF and reduced response to several viral peptides. Low CD4^+^ and CD8^+^ cell proliferation was observed both in DF and DHF; however, the lowest response was found in patients with hemorrhagic symptomatology. This could be associated with immune exhaustion observed in some studies on dengue [9, 41, 54, 55]. Exhausted T cells are characterized by specific markers such as PD-1, IL-7R, and ICOS, reduced capacity to secrete IFN-gamma, IL-2, or effector molecules (granzymes, perforin), and limited expansion capacity [41, 56].

The low levels of CD8^+^ T cells and reduced cytotoxic function observed in DHF patients may be related with kinetics of T cell responses on primary and secondary infection and not necessarily with the clinical outcome. During secondary heterologous DENV infection, an early and stronger T cell response was observed [9, 42]. It is also probable that the low levels of CD8^+^ cells are the consequence of previous activation that occurred early during infection and that negative feedback signals (IL-10, TGF-*β*) downregulated the activity of T cells and reduced their numbers. Therefore, we could be observing this phenomenon in a late phase after activation, when T cell numbers are reduced again. Other clues of higher activation of CD8^+^ cells are apparent increase in CD25 expression and lower frequency of perforin found by flow cytometry in DHF. This hypothesis is not contradictory to that of cell exhaustion, proposed previously; both include activation and downregulation phases. It is probable that DENV activates downregulatory signals (IL-10) to reduce the antiviral immune response as an evasion strategy to persist in the organism [50]. Notably, the clinical worsening of dengue patients could be associated with reduced activity of cytotoxic T cells induced by Treg cells [57].

Preservation of homeostasis after the activation of the antiviral cytotoxic response requires the development of regulatory T cells that are induced under the control of FOXP-3 [57]. A notable polarization of Treg response in DHF patients was indicated by the high proportion of CD3^+^CD25^+^ cells and the 200-fold increase in FOXP-3 transcription that was not found in DF patients. Although FOXP-3 expression is not exclusive of the Treg linage [58], expansion of Treg cells in acute dengue suggests that these cells suppressed the proliferative response of DENV-specific cytotoxic T cells, as what occurred *in vitro* and in a mouse model [59, 60].

By other side, our results show that a Th17 response was not involved in the pathogenesis of dengue in these patients; this is indicated by the low expression of *ROR-γ* and the lack of IL-17 both in DHF and DF patients. This is contrary to other studies that showed increased concentrations of IL-17 in dengue patients [61–63], including a study in Mexican patients that showed high levels of TH17 cells and IL-17 might be induced by PMA and ionomycin used to promote in vitro proliferation and differentiation of Th17 cells [63]. Reduced expression of ROR-*γ* in our study indicates that an intense downregulation process occurred in this gene associated with Th17 cells and IL-17 secretion. ROR-*γ* downregulation has been associated with T cell maturation in the thymus and differentiation of helper T cells in peripheric lymphoid organs; it is also related to several disorders, including infections. ROR-*γ* is downregulated by the presence of IL-10 in the microenvironment. Therefore, it is probable that reduced levels of ROR-*γ* is also consequence of FOXP3 expression and IL-10 secretion. In addition, ROR-*γ* is also susceptible to downregulation by GATA-3.

We also analyzed the presence of activation biomarkers on lymphocytes and other cells. CD40L expression was significantly lower in patients with DHF, but the proportion of CD25^+^ (associated with Tregs and other cells) was significantly higher. CD40L is involved in the secretion of IL-12 as well as in the activation and differentiation of Th1 cells. Reduced numbers of Th1 cells have been reported in patients with severe dengue [64] and are associated with the presence of Treg cells, production of IL-10, and downregulation of CD40L [65, 66]. We and others have found the association of IL-10 levels with dengue severity [22, 50, 64].

Although patients were clinically classified in DF or DHF, our results should be taken with some reserve because most patients have reduced platelet counts. Platelet counts < 100,000 cells/mm^3^ are indicative of high risk for hemorrhage, and almost all patients in our study have <10,000 cells/ml and any kind of hemorrhagic manifestations that indicates a severe hematological compromise. In addition, all patients were hospitalized; this reflects dengue complications, although patients classified as DF stay fewer days in hospitalization than DHF patients.

## 5. Conclusions

In this study, we analyzed the phenotype and functionality of immune cells in patients with dengue. Reduced amounts of functional cytotoxic CD8^+^ T cells and low expression of T-bet involved in Th1 differentiation were found in DHF patients that in contrast showed high levels of Th2 responses of CD4^+^ (GATA-3) and regulatory (Foxp3) T lymphocytes. These confluent characteristics determine the low antigenic response induced by viral peptides on immune cells of DHF patients and reveal the imbalance of Th1/Th2/Treg cells induced by dengue infection.

## Figures and Tables

**Table 1:** Characteristics of dengue virus peptides used in this study.

Peptide ID	Protein	Sequence	Position	Induced response	Reference
P1	E	FKNPHAKKQDVV	519-530	CD4^+^ and CD8^+^	Sánchez-Burgos et al., 2010
P2	E	RGARRMAIL	687-695	CD8^+^ and antibodies	Sánchez-Burgos et al., 2010
P3	E	DFGSVGGVL	699-707	Mostly antibodies	Sánchez-Burgos et al., 2010
P4	NS3	WITDFKGKTVW	1824-1834	CD4^+^^∗^	Zeng et al., 1996
P5	NS3	TPEGITPAL	1975-1983	CD4^+^ and CD8^+^	Livingston et al., 1995
P6	NS3	GTSGSPIVNR	1608-1617	CD8^+^	Friberg et al., 2011
P7	NS4a	ASIILEFFL	2199-2207	Low CD4^+^ and CD8^+^ and antibodies	Sánchez-Burgos et al., 2010
P8	NS4a	LRPASAWTL	2271-2279	Mostly antibodies	Sánchez-Burgos et al., 2010
P9	NS4a	CYSQVNPTTL	2337-2346	CD8^+^ and antibodies	Sánchez-Burgos et al., 2010
P10	NS4b	GSYLAGAGL	2469-2477	High CD4^+^ and CD8^+^	Sánchez-Burgos et al., 2010
P11	NS5	VIPMVTQIAMTDTTP	2826-2840	CD4^+^ and CD8^+^ and antibodies	Sánchez-Burgos et al., 2010
P12	NS5	YMWLGARFL	2967-1975	Mostly antibodies	Sánchez-Burgos et al., 2010
P13	NS5	SYSGVEGEGL	3003-3012	Low CD4^+^ and high CD8^+^	Sánchez-Burgos et al., 2010
P14	NS5	YFHRRDLRL	3257-3265	Mid CD4^+^ and high CD8^+^	Sánchez-Burgos et al., 2010

Most peptide sequences were obtained from Sánchez-Burgos et al. (2010). P4, P5, and P6 were selected from Zeng et al., Livingston et al., and Friberg et al., respectively [38–40]. Position refers to the first and last amino acid in the polyprotein. Peptides that induced IFN-*γ* production in mouse or human CD4 and CD8 cells were selected for the present study. ^∗^Some peptides that induced antibody production and low CD4 and CD8 cell activity were selected as controls of activation. ^∗∗^CD8 cells were not tested.

**Table 2:** Clinical and laboratory features of patients with dengue fever (DF) and dengue hemorrhagic fever (DHF).

Clinical finding	DF (*n* = 63)	DHF (*n* = 50)	*p* value
Pleural effusion or ascites^1^	0	1 (2%)	—
Tourniquet (+)^1^	2 (3%)	5 (10%)	—
Bleeding manifestations^1^	12 (19%)	34 (68%)	0.0001
Hematocrit^2^	39.75 ± 4.9%	37.8 ± 5.42%	—
Hemoglobin^3^	13.55 ± 5.42 g/dl	12.6 ± 1.87 g/dl	—
Creatinine^3^	0.88 ± 0.33 mg/dl	0.77 ± 0.26 mg/dl	0.2724
<0.6 mg/dl	7 (11%)	11 (22%)	0.0556
Albumin^2^	3.23 ± 0.54	2.97 ± 0.5	0.0046
<2.5 mg/dl	6 (9%)	8 (16%)	0.1989
2.5-3.4 mg/dl	32 (51%)	33 (66%)	0.0314
Platelet counts^2^ (cells/mm^3^)	67,660 ± 26,430	26,240 ± 18,180	0.0001
<25,000 cells/mm^3^	19 (30%)	27 (54%)	0.0005
25,000–49,000 cells/mm^3^	16 (25%)	17 (34%)	0.2146
50,000–99,000 cells/mm^3^	12 (19%)	6 (12%)	0.2408
>100,000 cells/mm^3^	17 (27%)	0	—
WBC count^2^, cells × 10^3^/*μ*l	5.246 ± 2.589	6.325 ± 3.511	0.2225
WBC differential counts^2^ (% of total cells)			
Lymphocytes	41.384 ± 13.232	32.5 ± 15.382	0.1149
MID cells	15 ± 6.976	9.833 ± 5.373	0.0508
Neutrophils	43.548 ± 14.063	57.333 ± 19.075	0.0696
Hepatic enzymes			
ALT^2^	114 ± 76 UI/ml	113 ± 107 UI/ml	0.9403
>80 UI/ml	37 (59%)	30 (60%)	0.4757
>200 UI/ml	6 (9%)	8 (16%)	0.1989
AST^2^	159 ± 128 UI/ml	178 ± 209 UI/ml	0.7119
>80 UI/ml	43 (68%)	40 (80%)	0.0756
>200 UI/ml	14 (22%)	15 (30%)	0.2590

^1^Number of patients with the symptom (and percentage). ^2^Mean value ± SD. ^3^Mean concentration ± SD. *p* values were determined by the Mann–Whitney *U* test for continuous variables and by the *χ*^2^ test for categorical variables; ^∗^*p* < 0.05 indicates significance between DF and DHF.

**Table 3:** Analysis of activation molecules on immune cells of dengue patients by flow cytometry.

	DF (*n* = 23)	DHF (*n* = 20)	*p* value
CD40L^+^	19.06 ± 13.54	4.02 ± 2.96	0.0009^∗^
CD45^+^	53.02 ± 16.09	35.73 ± 10.4	0.0004^∗^
CD69^+^	7.84 ± 4.15	9.79 ± 4.04	0.0987
CD25^+^	7.49 ± 3.49	18.36 ± 8.15	0.0001^∗^
CD3^+^CD40L^+^	3.19 ± 1.98	1.65 ± 1.29	0.0053^∗^
CD3^+^CD45^+^	36.46 ± 12.75	32.21 ± 9.15	0.8184
CD3^+^CD69^+^	4.94 ± 2.01	4.54 ± 1.31	0.8901
CD3^+^CD25^+^	1.24 ± 0.81	3.01 ± 2.02	0.0018^∗^

Data are from flow cytometry experiments shown in Figure 3; numbers represent percentages. *p* values were determined by the Mann–Whitney *U* test; ^∗^mean significance between DF and DHF, *p* < 0.05.

## Data Availability

The clinical and laboratory data used to support the findings of this study are restricted by the Local Committees of Ethics and Research in Health of the Mexican Institute of Social Security, in order to protect patient privacy. Data are available from the corresponding author upon request for researchers who meet the criteria for access to confidential data.
